# Interferon alpha as antiviral therapy in chronic active Epstein-Barr virus disease with interstitial pneumonia - case report

**DOI:** 10.1186/s12879-018-3097-6

**Published:** 2018-04-20

**Authors:** Jacek Roliński, Ewelina Grywalska, Aleksandra Pyzik, Michał Dzik, Violetta Opoka-Winiarska, Agata Surdacka, Maciej Maj, Franciszek Burdan, Michał Pirożyński, Piotr Grabarczyk, Elżbieta Starosławska

**Affiliations:** 1 Department of Clinical Immunology and Immunotherapy, Medical University of Lublin, Chodzki 4a Street, 20-093 Lublin, Poland; 2 St. John’s Cancer Centre, Jaczewskiego 7 Street, 20-090 Lublin, Poland; 3 Department of Paediatric Pulmonology and Rheumatology, Medical University of Lublin, Gebali 6 Street, 20-093 Lublin, Poland; 4 Department of Anaesthesiology and Critical Care Medicine, Postgraduate Medical School, Marymoncka 99/103 Street, 01-813 Warsaw, Poland; 5 Institute of Haematology and Transfusion Medicine, Indihri Gandhi 14 Street, 02-776 Warsaw, Poland

**Keywords:** Chronic active Epstein-Barr virus disease, Epstein-Barr virus, Interferon alpha, Interstitial pneumonitis

## Abstract

**Background:**

Chronic active Epstein-Barr virus (EBV) disease (CAEBV) is defined as a severe, progressive lymphoproliferative disorder associated with active EBV infection persisting longer than 6 months and developing in patients without recognised immunodeficiency. Rarely, interstitial pneumonitis (IP) occurs as a serious complication in CAEBV patients. The standard therapeutic regimen for IP in CAEBV has not yet been defined. Although interferon alpha (IFN-alpha) is known to suppress viral DNA replication by affecting its basal promoter activation process, it is rarely used in CAEBV patients.

**Case presentation:**

A 22-year-old Caucasian woman, diagnosed with CAEBV 1.5 years earlier, was admitted to the Immunology Clinic due to a 4-week history of productive cough, fever and general weakness. Cultures of blood, urine and sputum were negative, but EBV DNA copies were found in the sputum, whole blood, isolated peripheral blood lymphocytes as well as in the blood plasma. Cytokine assessment in peripheral blood revealed the lack of IFN-alpha synthesis. Disseminated maculate infiltrative areas in both lungs were observed on a computed tomography (CT) chest scan. The patient was not qualified for the allogeneic hematopoietic stem cell transplantation (allo-HSCT) due to the risk of immunosuppression-related complications of infectious IP. Inhaled (1.5 million units 3 times a day) and subcutaneous (6 million units 3 times a week) IFN-alpha was implemented. To the best of our knowledge, this was the first documented use of inhaled IFN-alpha in a patient with CAEBV and concomitant IP. Patient’s status has improved, and she was eventually qualified to allo-HSCT with reduced conditioning. Currently, the patient feels well, no EBV was detected and further regression of pulmonary changes was documented.

**Conclusions:**

CAEBV should be considered in patients who present with interstitial lung infiltration and involvement of other organs. Although more promising results have been obtained with allo-HSCT, inhaled IFN-alpha may also be a therapeutic option in patients with CAEBV and a concomitant IP.

## Background

Detection of Epstein-Barr virus (EBV) infection, acute or chronic, may be an important finding during differential diagnosis of autoimmune systemic disorders. Primary EBV infection manifests clinically as mononucleosis, a self-limiting disease lasting no longer than 2-3 weeks [1, 2]. Chronic active Epstein-Barr virus disease (CAEBV) is defined as a severe, progressive lymphoproliferative disorder associated with active EBV infection persisting longer than 6 months and developing in patients without recognised immunodeficiency [3]. This life-threatening condition is characterised by markedly elevated levels of antibodies against EBV or EBV deoxyribonucleic acid (DNA) in the blood and EBV ribonucleic acid (RNA) or protein in lymphocytes in tissues. Most patients present with fever, hepatic dysfunction, splenomegaly, lymphadenopathy, and thrombocytopenia. Other features, found in more than 10% of patients, include hepatomegaly, anaemia, rash, oral ulcers, haemophagocytic syndrome, coronary artery aneurysms, liver failure and lymphoma. Rarely, interstitial pneumonitis (IP) occurs as a serious complication of CAEBV [4–6]. Standard therapeutic regimen for CAEBV-associated IP has not yet been defined. Although interferon alpha (IFN-alpha) is known to suppress viral DNA replication by affecting its basal promoter activation process, it is rarely used in patients with this condition. We present the case of a 22-year-old woman with CAEBV and IP, who due to treatment with IFN-alpha, could be eventually qualified for a successful allo-HSCT procedure.

## Case presentation

A 22-year-old Caucasian woman, diagnosed with CAEBV 1.5 years earlier, was admitted to the Immunology Clinic due to a 4-week history of productive cough, fever and general weakness. Previously, the patient had been receiving antibiotics for a month in an outpatient setting, but no significant improvement was observed [7].

The patient had been diagnosed with infectious mononucleosis at the age of 17 years, with resultant significant deterioration of her status lasting for 6 months. She was eventually hospitalized due to leucopoenia, anaemia, splenomegaly, lymphadenopathy and liver dysfunction. Antinuclear antibody titre was 1:80, the ENA (extractable nuclear antigen) panel and antineutrophil cytoplasmic antibody (ANCA) yielded negative results, and C3, C4 complement concentrations were normal.

Despite extensive diagnostic workup, including, among others, bone marrow and liver biopsy, a diagnosis has never been established.

During subsequent two years, the patient was rehospitalized a few more times, and periodically received treatment with granulocyte colony-stimulating factors (G-CSF) and corticosteroids. At the age of 20 years, the woman underwent splenectomy due to hypersplenism and splenomegaly. Six months later she was diagnosed with CAEBV, according to the Okano criteria 2005 [8] i.e.: 1) persistent or recurrent infectious mononucleosis-like symptoms; 2) an unusual pattern of EBV antibodies with elevated anti-VCA (viral capsid antigen) and anti-EA (early antigen), or detection of the EBV genome in affected tissues including the peripheral blood; and 3) chronic illness that cannot be explained by any other known disease processes at the time of diagnosis. In situ hybridisation (ISH) staining of spleen specimens revealed expression of EBV latent membrane protein 1 (LMP1) and EBV-encoded small RNAs (EBERs) in T lymphocytes (T-cell type infection). After these findings, the patient was put on immunostimulatory treatment with thymus peptides. The woman has never smoked or used other tobacco products, and has never been exposed to inhalant irritants. She also had no family history of pulmonary malignancies or other lung diseases.

At the time of admission to the Immunology Clinic, the patient had no fever. Neither respiratory failure or cyanosis were observed. No abnormalities were found on physical examination, except for axillary and inguinal lymph node enlargement (up to 1 cm), and single bilateral wheezes audible above the region corresponding to middle and lower lung fields.

Patient’s haemoglobin concentration was 9.9 g/dl and her WBC (white blood cells) amounted to 6300/μl, with 20.5% of lymphocytes, 66.6% of neutrophils and 6.5% monocytes. The other parameters of the blood were as follows: platelets 607,000/μl, ESR (erythrocyte sedimentation rate) 120 mm/h, CRP 8.71 mg/dl, aspartate aminotransferase 126 u/l, alanine aminotransferase 88 u/l, total bilirubin 0.14 mg/dl and D-dimers 1817 ng/ml. The results of kidney function tests and electrolyte levels were normal.

**Fig. 1:**
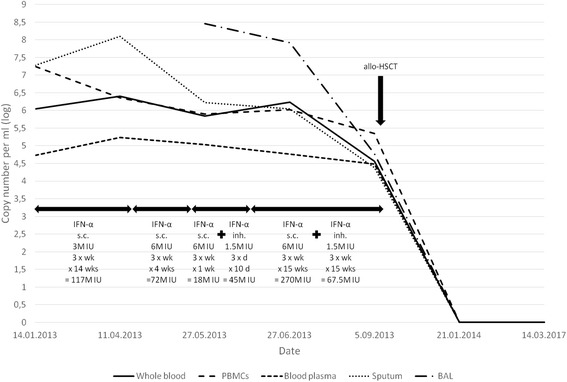
EBV DNA copy number (log) before, during and after treatment

Bacterial (aerobic and non-aerobic) and fungal cultures were carried out under standard conditions, and yielded no pathogens. Also polymerase chain reaction (PCR)-based testing for genetic material of hepatitis C virus (HCV), hepatitis B virus (HBV), human immunodeficiency virus (HIV), herpes simplex virus 1 and 2 (HSV-1 and -2), cytomegalovirus (CMV), human papillomavirus (HPV), parvovirus B19, influenza virus, Borrelia burgdorferi, Chlamydia trachomatis, Chlamydia pneumoniae, Mycobacterium tuberculosis, Toxoplasma gondii, Ureaplasma spp. and Listeria spp. yielded no positive results. EBV DNA copies were found in the sputum, whole blood, isolated peripheral blood lymphocytes as well as in the blood plasma (Fig. 1). Other potential pathogens causing the condition were excluded.

Serum cytokines i.e.: granulocyte-macrophage colony-stimulating factor (GM-CSF), IFN-gamma, interleukin (IL)-1-beta, IL-2, IL-4, IL-5, IL-6, IL-7, IL-8, IL-10, IL-12 (p70), IL-13, and tumour necrosis factor (TNF)-alpha were measured by the use of the Multiplex MAP high sensitivity human cytokine panel (Millipore), following the manufacturer’s instructions, similar as described previously by Cohen et al. [6]. The results were as follows: GM-CSF: 4639.97 pg/mL, IFN-gamma: > 2500 pg/mL, IL-1-beta: 23.72 pg/mL, IL-2: 13.91 pg/mL, IL-4: 6.16 pg/mL, IL-5: 174.77 pg/mL, IL-6: > 750 pg/mL, IL-7: 75.19 pg/mL, IL-8: 625.08 pg/mL, IL-10: > 6000 pg/mL, IL-12 (p70): 1978.23 pg/mL, IL-13: 27.65 pg/mL, TNF-alpha: > 1750 pg/mL. Concentration of IFN-alpha was determined with Human IFN-alpha ELISA kit (Thermo Fischer Scientific) and Human IFN-alpha ELISA Kit (R&D Systems). The concentration of IFN-alpha was assessed in serum, saliva, bronchoalveolar lavage (BAL) and peripheral blood mononuclear cells (PBMCs) culture supernatant. The assays yielded no detectable levels of this cytokine.

**Fig. 2:**
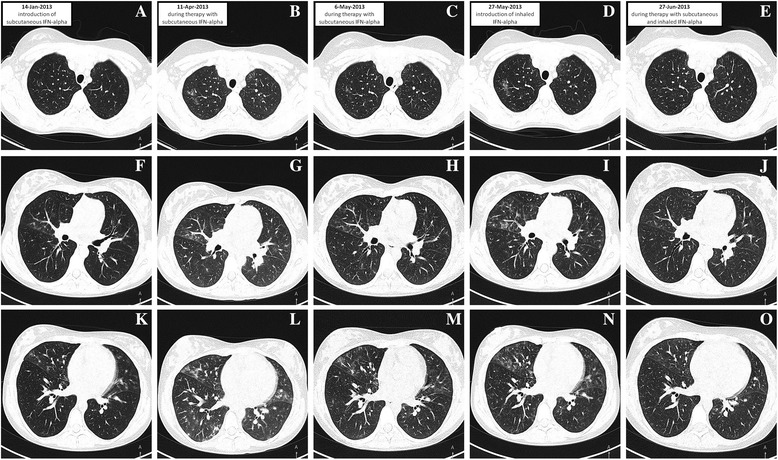
Consecutive CT scans obtained from the patient: AFK – before introduction of IFN-alpha (14-Jan-2013), BGL – during treatment with subcutaneous IFN-alpha (11-Apr-2013), CHM – during treatment with subcutaneous IFN-alpha (6-May-2013), DIN – before introduction of inhaled IFN-alpha (27-May-2013), EJO – during treatment with subcutaneous IFN-alpha and inhaled IFN-alpha (27-Jun-2013)

Clear lung fields and normal cardiac and mediastinal silhouettes could be observed on chest radiograph. However, disseminated maculate infiltrative areas in both lungs, more prevalent on the right side, were observed on a CT chest scan (Fig. 2, AFK scans). Abdominal ultrasound revealed enlarged liver with highly heterogeneous echogenicity, but without evident focal changes, enlarged (up to 10 mm) paraaortic lymph nodes, small volume of free fluid in minor pelvis and post-splenectomy status. No other abnormalities were found. The patient was disqualified as for the allogeneic hematopoietic stem cell transplantation (allo-HSCT) due to the risk of immunosuppression-related complications of infectious IP [3, 4].

Long-term treatment with subcutaneous IFN-alpha was implemented (3 million units, 3 times a week) [9]. However, patient’s condition deteriorated three months later: April 2013 she presented with fever, persistent cough and was in generally poor physical health (Fig. 2, BGL). The dose of IFN-alpha was escalated to 6 million units 3 times a week, but still with no significant clinical improvement. CT performed one month later (May 2013) revealed only partial regression of pulmonary changes (Fig. 2, CHM). Therefore, the patient was referred to the Pulmonology Clinic. She had 67.81% of lymphocytes with 94.19% CD3 T cells, CD4/CD8 ratio equal to 11.35 (Table 1). Owing to poor response to previous treatment, inhaled IFN-alpha (1.5 million units 3 times a day) was added (Fig. 2DIN). To the best of our knowledge, this was the first documented use of the inhaled IFN-alpha in a patient with CAEBV and concomitant IP. Patient’s status has finally improved after 10 days of treatment.

**Table 1:** Changes in basic laboratory parameters over the treatment time

Parameter	Date				
	14-Jan-2013	11-Apr-2013	27-May-2013	27-Jun-2013	5-Sep-2013
WBC (× 10^9^/L)	6.3	4.27	5.18	4.43	8.48
Lymphocytes (× 10^9^/L)	1.29	0.7	0.61	0.85	2.74
Neutrophils (× 10^9^/L)	4.2	2.82	3.23	2.88	4.8
Monocytes (× 10^9^/L)	0.41	0.48	0.83	0.31	0.29
Hgb (g/L)	99	96	98	102	116
Hct (%)	31	28	29.8	32	33.4
PLT (× 10^9^/L)	607	532	428	414	313
ALT (U/L)	88	97	48	30	37
AST (U/L)	126	117	86	69	40
ESR (mm/h)	120	120	120	39	24

Furthermore, partial regression of pulmonary changes was observed on a CT scan obtained one month later (Fig. 2EJO, June 2013). Figure 1 presents changes in EBV DNA copy numbers during the treatment.

The patient was qualified to allo-HSCT with reduced conditioning, which was carried out 6 months later (December 2013). Currently, the patient feels well, no EBV was detected in whole blood, blood plasma, PBMCs and sputum, and further regression of pulmonary changes was documented.

## Discussion and conclusions

Interstitial pneumonia is a diffused chronic interstitial lung disease associated with inflammation and/or fibrosis of the alveolar walls [10]. Three pulmonary manifestations associated with EBV infection: hilar/mediastinal lymphadenopathy, pleural effusion, and IP have been described in literature [5]. EBV-associated IP may be triggered by drugs, occupational exposure, hypersensitivity reactions and infections; some cases are, however, idiopathic [11]. Clinical presentation is dominated either by inflammatory changes or by fibrosis. Concomitant IP is rarely observed during the course of in T-cell CAEBV, but still may develop in ca. 5% of patients with the latter condition [11, 12]. Symptoms are not specific, and include chronic cough, tachypnoea, dyspnoea and fever [10–14]. Only few reports describe IP as a complication of chronic active EBV infection in immunocompetent patients.

Successful outcomes in IP depend mainly on appropriate identification of an underlying pathology [10–12]. IFN-alpha is a recognised immunomodulatory therapy to suppress viral replication by inhibiting basal transcription processes. IFN-alpha was shown to be effective in patients with CAEBV [15, 16]. Sakai et al. reported the application of IFN-alpha to patients with CAEBV resulting in unremarkable suppression of lymphocyte proliferation [16]. In the case studied by Mitsui et al., treatment with IFN-alpha temporarily attenuated clinical symptoms: fever, skin and mucosal lesions, but the patient eventually developed pulmonary effusion and died of cardiac insufficiency [7]. A number of other therapies have been tried for CAEBV including antiviral agents: acyclovir, ganciclovir, immunomodulators: interleukin (IL)-2, chemotherapy: etoposide, corticosteroids, cyclosporine, and EBV-specific cytotoxic T cells (CTLs) [2–4, 6, 7, 16–20]. Recently, more promising results have been obtained with HSCT. HSCT may eliminate EBV-infected cells, reconstitute EBV-specific cellular immunity, and trigger a graft-versus-tumour effect. However, the procedure is associated with high risk of transplantation-related complications and the 5-year survival rate was only 53% [6].

5-year survival rate in patients with CAEBV is estimated at 59% [2–4]. IP is a serious and life-threatening complication of CAEBV [2–4]. In our patient, the disease was diagnosed late, 3 years after primary EBV infection. The course of the disease was then complicated by IP. To the best of our knowledge, there is no published information about the inability to synthesize IFN-alpha and a link between this pathology and CAEBV with IP development. Considering lack of IFN-alpha and the fact that the patient failed to be qualified for HSCT at any transplantation centre, we have implemented the treatment which has been previously used in some centres [6]. During initial 14 weeks, long-term treatment with subcutaneous IFN-alpha was implemented (3 million units, 3 times a week), which corresponded to a cumulative dose of 117 million units. In April 2013, the dose was escalated to 6 million units 3 times a week; this regimen was continued for 4 weeks (72 million units overall). Altogether, 189 million units of IFN-alpha were administered subcutaneously. In May 2013, we continued subcutaneous IFN-alpha (6 million units 3 times a week) and added inhaled one (1.5 million units 3 times a day). We prepared the inhaled IFN-alpha solution, using Roferon®-A (Interferon alfa-2a 3 MIU solution for injection, Roche). Using a pneumatic inhaler, 2 ml of the mixture were delivered over 5 min. Each 2-ml dose contained Roferon®-A – half of a labelled dose (3 million IU in 0.5 ml), i.e. 1.5 million IU in 0.25 ml, mixed with 1.75 ml 0.9% physiological saline. To the best of our knowledge, this route of administration has not been used thus far. Already after 10 days of treatment (after a cumulative dose of 45 million units of inhaled IFN-alpha and 18 million units of subcutaneous IFN-alpha), an improvement was observed, but the treatment was continued for another 15 weeks (1.5 million units 3 times a week, altogether 67.5 million units of inhaled IFN-alpha and 270 million units of subcutaneous IFN-alpha), till 10 September 2013, when due improvement of clinical status, the patient was qualified for HSCT. Overall, the patient received 112.5 million units of inhaled IFN-alpha and 477 million units of subcutaneous IFN-alpha). We summarized the treatment phases on Fig. 1, along with DNA EBV copy numbers at various timepoints. Obtained results revealed that IFN-alpha (subcutaneous and inhaled) was effective in controlling the inflammatory process, and the patient could be eventually qualified for a successful HSCT.

In conclusion, the CAEBV disease should be considered in the differential diagnosis of patients with systemic symptoms. IP is life-threatening condition which may be caused by infection with EBV. Inhaled IFN-alpha in combination with subcutaneous IFN-alpha might be a therapeutic option in patients with CAEBV and concomitant IP and should be investigated more thoroughly.

## Abbreviations

allo-HSCT: Allogeneic hematopoietic stem cell transplantation
ALT: Alanine aminotransferase
ANCA: Antineutrophil cytoplasmic antibody
AST: Aspartate aminotransferase
BAL: Bronchoalveolar lavage
CAEBV: Chronic active Epstein-Barr virus disease
CMV: Cytomegalovirus
CRP: C reactive protein
CT: Computed tomography
CTLs: Cytotoxic T cells
DNA: Deoxyribonucleic acid
EA: Early antigen
EBERs: EBV-encoded small RNAs
EBV: Epstein-Barr virus
ENA: Extractable nuclear antigen
ESR: Erythrocyte sedimentation rate
G-CSF: Granulocyte colony-stimulating factors
GM-CSF: Granulocyte-macrophage colony-stimulating factor
HBV: Hepatitis B virus
Hct: Haematocrit
HCV: Hepatitis C virus
Hgb: Haemoglobin
HIV: Human immunodeficiency virus
HPV: Human papillomavirus
HSCT: Hematopoietic stem cell transplantation
HSV-1 and − 2: Herpes simplex virus 1 and 2
i.v.: Intravenous
IFN-alpha: Interferon alpha
IL: Interleukin
IP: Interstitial pneumonitis
ISH: In situ hybridisation
LMP1: Latent membrane protein 1
PBMCs: Peripheral blood mononuclear cells
PCR: Polymerase chain reaction
PLT: Blood platelets
RNA: Ribonucleic acid
RT-PCR: Reverse transcriptase polymerase chain reaction
TNF: Tumour necrosis factor
VCA: Viral capsid antigen
WBC: White blood cells
